# Intake Modalities of Amino Acid Supplements: A Real-World Data Collection from Phenylketonuria Patients

**DOI:** 10.3390/nu16050669

**Published:** 2024-02-27

**Authors:** Albina Tummolo, Rosa Carella, Pasquale Carone, Giulia Paterno, Donatella De Giovanni

**Affiliations:** 1 Division of Metabolic and Genetic Diseases, Children Hospital Giovanni XXIII, Azienda Ospedaliero-Universitaria Consorziale, 70126 Bari, Italy; 2 Department of Precision and Regenerative Medicine and Ionian Area, University of Bari “Aldo Moro”, 70126 Bari, Italy

**Keywords:** phenylketonuria, phenylalanine, amino acid mixtures, prescription adherence, intake modalities, dietary control, metabolic control

## Abstract

Background: To achieve a normal nutritional status, patients suffering from phenylketonuria (PKU) are typically prescribed amino acid (AA) supplements with low or no phenylalanine (Phe) content. Studies evaluating patient preferences regarding the intake modalities of AA supplements are limited. This study aimed to collect real-world data regarding prescription adherence and intake modalities of AA supplements reported by PKU patients while monitoring metabolic control. Methods: This cross-sectional study included 33 PKU patients (16 female and 17 male) with a mean age of 27.2 years. Questionnaires were provided to assess information on AA supplement intake, such as prescription adherence rate, frequency and timing of administration, supplement formulation, and combination with food or drinks. Plasma phenylalanine levels were monitored during the study period. Results: 51.5% (*n* = 17) of patients reported to lay within an adherence range of 75–100%. The majority of patients consumed AA supplements twice daily, with breakfast (87.9%) and afternoon snacks (51.5%). Powder supplements were most commonly used (72.7%) and often combined with milk and/or fruit juices (45.4%). Conclusions: Despite the known concerns related to treatment compliance among PKU adolescents and adults, most of the study participants reported a high level of adherence to AA supplement prescription. The personalized dietary regimens followed by the patients included in the current study represent a treatment approach that might be worth trying in non-compliant patients.

## 1. Introduction

Phenylketonuria (PKU) is an inherited disorder caused by mutations of the phenylalanine hydroxylase (PAH) gene, occurring in approximately 1 over 15,000 individuals but with high incidence variability among countries [1,2,3].

The PAH enzyme—together with tetrahydrobiopterin (BH4), molecular oxygen, and iron—is involved in the metabolization of phenylalanine (Phe), assuming the role of converting it into tyrosine (Tyr). Therefore, the PAH enzyme or BH4 cofactor deficiencies induce increased Phe levels and decreased Tyr concentrations in the blood and brain, eventually causing a spectrum of disorders that include classic PKU, mild PKU, and mild hyperphenylalaninemia (HPA) [1,3,4].

If left untreated, classic PKU can lead to progressive intellectual disabilities, entailing psychiatric and neurological symptoms, whereas mild PKU and mild HPA are associated with lower risks of experiencing cognitive impairments, even in the absence of treatment [1,3].

For the management of PKU patients, a low-Phe diet combined with amino acid (AA) supplementation allows for the prevention of neurological impairments by maintaining plasma Phe levels below the safe limit of 360 µmol/L and 600 µmol/L in patients younger and older than 12 years old, respectively [5]. However, to obtain such results, PKU treatment should start as early as possible and be maintained over time [6]. Unfortunately, if parental supervision ensures high levels of treatment adherence during childhood, adolescents often stop following dietary restrictions [1,6,7]. Moreover, despite the risk of late-onset neurological deficits, adults also frequently discontinue diet therapy, running a further risk of suffering from a more systemic disease [8,9,10,11]. Accordingly, a systematic review identified age as one of the major factors associated with poor treatment adherence, highlighting a gradual decline in metabolic control from adolescence onward [12].

Along with age, several other factors contribute to treatment discontinuation, as identified by several questionnaire-based studies. According to the results of a multicenter survey, patient-reported factors include social difficulties, unpleasant organoleptic properties of AA supplements, as well as discomfort and embarrassment when taking supplements away from home [13]. Additionally, a review that focused on treatment adherence in PKU patients revealed further elements with an impact on compliance, which include limited patient awareness of the disease, inadequate family support, emotional stress, and financial burden [14]. While difficulties in following the PKU diet have been extensively reported, less is known about adolescent and adult PKU patient preferences regarding AA supplement consumption and whether their assumption is related to natural protein intake compliance.

In this context, the objective of this study was to collect real-world data from adolescent and adult PKU patients regarding treatment adherence, modality, and timing of AA supplement intake and to relate them to blood Phe levels as a measure of the achieved metabolic control.

## 2. Materials and Methods

This cross-sectional study was conducted at the Department of Metabolic Diseases, Clinical Genetics and Diabetology of the Giovanni XXIII Children’s Hospital (Azienda Ospedaliero-Universitaria Consorziale, Bari, Italy). In accordance with the institution’s regulations, ethical approval was not sought since the data were retrieved from clinical databases and patient-reported questionnaires, not including subject-identifying or sensitive data. Nevertheless, ethical considerations were made with respect to the principles for research on human subjects as outlined in the Declaration of Helsinki.

Data were collected from 33 patients who fulfilled the following inclusion criteria: age ≥ 12 years, diagnosed with PKU via newborn screening, regularly followed up, and undergoing diet therapy and AA supplementation.

Two PKU expert dietitians provided patients with questionnaires aimed at gathering information about AA supplement intake, which were categorized into five items: adherence to the prescribed treatment, pooled in three ranges (25–49%, 50–74%, and 75–100%); type of supplement, including amino acid mixtures (AAMs), long-chain neutral amino acids (LNAAs), and glycomacropeptide (GMP); formulation type (powder, liquid, and micro-tablets); intake modality (with water, fruit juice, milk, milk-based beverage, alone, or in combination with other foods); and intake frequency and timing. All patients were advised to consume supplements three or four times a day, depending on the amount of synthetic protein prescribed, at main meal times.

Blood Phe levels were monitored during the entire study period. Plasma phenylalanine levels were determined as follows. To separate plasma, 2 mL of blood collected in lithium heparin vacutainer tubes (BD-Plymouth.PL6 7BP, Plymouth, United Kingdom) were centrifugated using an MPW-150R centrifuge (MPW-MED Instruments, Warszawa, Poland) at 12,000 rpm and 7 °C, within 60 min after blood sampling. To determine plasma phenylalanine levels, high-performance liquid chromatography (HPLC) was performed using a Biochrom 30+ instrument (Erreci Srl, Milan, Italy). Lithium column and post-column derivatization using ninhydrin were employed. Absorbance was measured at wavelengths of 570 and 440 nm. Analytical data were processed using EZChrom chromatography software version A.04.10 (Agilent Technologies, California, United States). The pre-analytical working conditions were as previously described [15].

Regarding descriptive statistics, frequency distributions of categorical variables were analyzed as a count and percentage, while continuous variables were described as a count, mean, and standard deviation (SD). A one-way ANOVA followed by a Bonferroni post hoc test was performed to compare blood Phe levels associated with the different treatment-adherence ranges. Statistical significance was set at *p* < 0.05, and all statistical analyses were performed using SAS software version 9.4 (SAS Institute Inc., Cary, NC, USA).

## 3. Results

The 33 participants included in the study had a mean age of 27.2 years (SD = 10.3; range 12–44). Of these, 51.5% (*n* = 17) were male, and 48.5% (*n* = 16) were female. As also reported in Table 1, 87.9% (*n* = 29) of patients suffered from classic PKU, 9.1% (*n* = 3) were affected by mild PKU, and only 1 patient had a BH4 cofactor deficiency (dihydropteridine reductase deficiency).

As regards treatment adherence, all patients reported consuming AAMs. Slightly more than half of them (51.5%, *n* = 17) declared to fall within the 75–100% adherence range, while the remaining patients were distributed as follows: 8 (24.2%) in the range of 50–74% and 8 (24.2%) in the 25–49% range.

**Figure 1:**
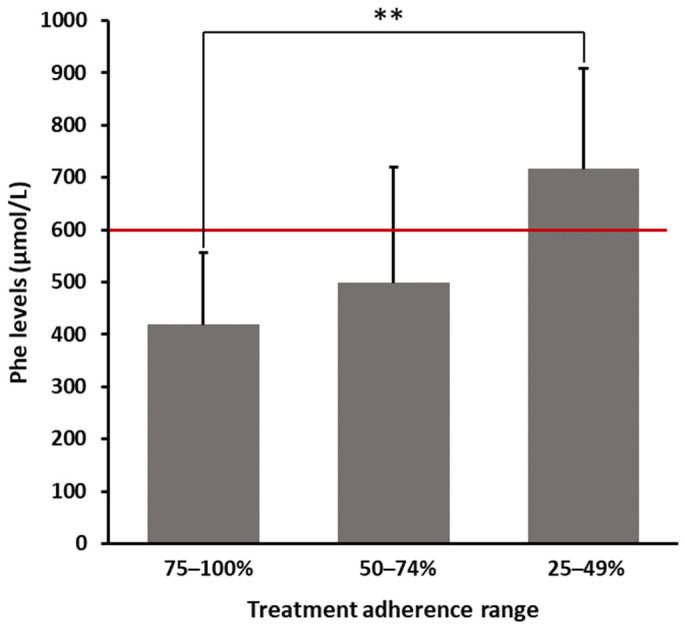
Blood Phe levels stratified according to treatment adherence range. Bars represent the means ± SD. Red line indicates the threshold (600 μmol/L) of safe Phe levels for patients aged ≥12 years. Phe = phenylalanine; SD = standard deviation; ** *p* < 0.01.

As shown in Figure 1, patients declaring a 75–100% adherence range had an average Phe value of 418.65 ± 137.23 µmol/L. In contrast, patients who claimed having not strictly followed prescriptions (25–49% adherence) showed significantly higher average levels of Phe (716.80 ± 191.97 µmol/L), exceeding the threshold limit of 600 µmol/L. Patients within an intermediate range of adherence (50–74%) showed a mean Phe value of 498.75 ± 221.66 µmol/L. As reported in Table 2, even after multiplicity adjustments, the difference in blood Phe levels between patients strictly and not strictly following prescriptions was statistically significant (*p* = 0.0037).

**Figure 2:**
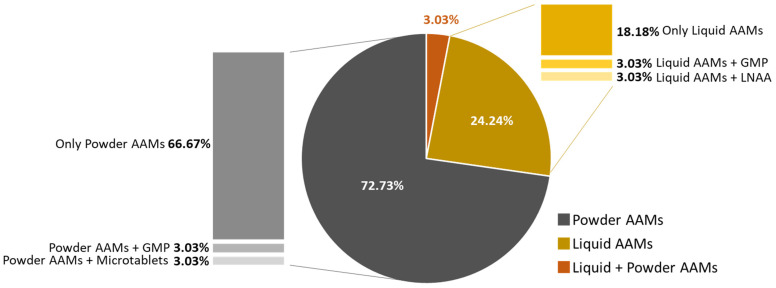
Patient preferences regarding supplement formulations. AAMs = amino acid mixtures; LNAA = long-chain neutral amino acids; GMP = glycomacropeptide.

Among all patients, 72.7% (*n* = 24) reported using powder formulations, 24.2% (*n* = 8) preferred liquid formulations, and only 1 patient (3.0%) consumed both types of formulations. Along with either powder or liquid AAMs, two patients also consumed GMP, one LNAA, and one micro-tablets (Figure 2).

Frequency distributions of the different intake modalities are reported in Table 3. Specifically, 45.4% (*n* = 15) combined the supplement with fruit juice, milk, or milk-based beverages, 21.2% (*n* = 7) consumed the supplement with water, and 9.1% (*n* = 3) took the supplement with other food, like fruit, free-protein biscuits, and syrup, by shaking them together in the same container. Three patients consumed the supplement using multiple methods, including alternating between water and food, between water and fruit juice, milk or milk-based beverages, and between food and fruit juice, milk, and milk-based beverages. A supplement alone was taken by 15.2% (*n* = 5) of the patients.

Regarding the frequency and timing of supplement consumption, the majority of patients (51.5%, *n* = 17) consumed supplements twice daily, 33.3% (*n* = 11) took supplements three times a day, and 9.1% (*n* = 3) consumed them once daily. Only two patients (6.1%) reported taking supplements four times a day.

Supplement consumption was spread over the day, with 87.9% (*n* = 29) of the patients taking them at breakfast. Given the multiple daily intakes, the remaining ones were distributed between a morning snack (*n* = 7), lunch (*n* = 8), an afternoon snack (*n* = 17), dinner (*n* = 5), and after dinner (*n* =12).

## 4. Discussion

The combination of a protein-restricted diet and AA supplementation is regarded as an effective treatment for patients with PKU [6]. Nevertheless, the transition from childhood to adolescence, and then to adulthood, represents a critical period hindering treatment compliance. Indeed, such transitions are typically associated with high rates of diet discontinuation and/or loss of follow-up [1,6,7,13].

By analyzing a sample of adolescents and adults with PKU in the present study, it was observed that nearly 76% of patients reported a medium/high level of prescription adherence. As expected, patients taking more supplements showed a higher degree of low-protein diet compliance, as demonstrated by the lower average values of Phe levels. Interestingly, the difference in blood Phe levels between patients who strictly followed prescriptions and those who did not strictly follow prescriptions was statistically significant. Therefore, the degree of supplementation compliance was coherent with blood Phe levels and, thus, the achieved metabolic control. Previous studies have reported highly variable percentages of patients complying with supplement prescriptions, which span from 42% [13] to percentages exceeding 80% [16], thus representing a value more in line with the findings presented herein. 

A surprisingly high proportion of patients included in this study (over 72%) preferred powder formulations to other options. According to the literature, factors like unpleasant taste and large-volume formulations—typical features of powder formulation—have been reported as disincentives to consume AA supplements [7,13,14], whereas single-dose formulations, avoiding the need to weigh or measure, are considered as positive features to overcome non-adherence issues [14]. Accordingly, a randomized cross-over study showed that the use of tablet formulations is associated with higher levels of treatment adherence (90%) when compared to the use of powder formulations (65%) in the same patients [17]. The different results that emerged in this study might be partially explained by the frequent observation of PKU subjects exhibiting skepticism towards new tastes and consistencies of both natural and medical food. Indeed, recent research has reported a high prevalence of neophobia among children and adults who had to follow a restricted diet since birth [18,19]. Therefore, as powder represents the most commonly used formulation during infancy and childhood, patients may have decided to continue with the same formula later in life, possibly to reconstitute a milk-like beverage. 

Another interesting observation of the present investigation regards the substantial proportion of patients preferring to consume supplements at breakfast or with morning/afternoon snacks rather than at lunch and/or dinner, which might also explain the way they were preferentially taken (i.e., with fruit juice, milk, or milk-based beverage). Indeed, avoiding supplement consumption at main meals may be attributed to their unpleasant odor and taste, eventually leading patients to associate their consumption with sugar-rich food. In this regard, several studies have reported an improved palatability of both GMP and/or LNAA as a way to promote a more regular supplement intake throughout the day [20,21,22]. In this study, intake habits reported by patients assuming GMP or LNAA did not differ from others, probably due to the small proportion of patients (3 out of 33) also using this formulation. 

Despite the abovementioned observations, it is worth noticing that official guidelines recommend taking supplements in conjunction with main meals as a strategy to ensure a satisfactory balance of protein and calories. Moreover, to minimize daily fluctuations in blood Phe levels and to reduce the oxidation of L-amino acids, it is advised to divide the prescribed amount of supplements into at least three equal portions and to consume them throughout the day [5,23,24,25,26]. 

Given the complexity of such a dietary regimen, adolescents and adults should be continuously supported and encouraged to strictly comply with official guideline recommendations and to maintain their blood Phe levels within optimal ranges. When failing to achieve this condition, alternative strategies such as meal composition optimization might be pursued. For instance, the caloric content of a meal, including supplement consumption, should be devised taking into account the high protein content of such an association. In wider terms, to prevent potential treatment failures and outcomes from worsening, the prescribed dietary regimen should fit the individual’s needs. 

Limitations of this study include the lack of long-term data and a possible selection bias, as only patients regularly attending the center—and thus possibly being more compliant—were investigated. The main novelty of this study is that, unlike other reports, it provides real-world data on patient preferences regarding supplement intake modalities, suggesting that therapy discontinuation in adolescent and adult PKU patients might be prevented by proposing individualized treatments capable of improving metabolic control and reducing long-term sequelae. From a future perspective, it would be extremely interesting to investigate the relationship between blood Phe levels and the frequency and timing of supplement administration. 

## 5. Conclusions

Despite the known concerns related to treatment compliance among PKU adolescents and adults, most of the study participants reported a high level of adherence to AA supplements prescription. The dietary regimens followed by the patients included in the current study—specifically envisioned and continuously adjusted to fit individual’s needs—represent a treatment approach that might be worth trying in non-compliant patients, eventually improving metabolic control and avoiding late-onset PKU-related complications. 

## Figures and Tables

**Table 1:** Patients’ characteristics.

Characteristic		Statistics ^1^
Age		27.2 (10.3)
Sex	Male	17 (51.5)
	Female	16 (48.5)
PKU Type	Classic	29 (87.9)
	Mild	3 (9.1)
	BH4 cofactor deficiency	1 (3.0)

^1^ Statistics are: mean (SD) for age; N (%) otherwise; PKU = phenylketonuria; SD = standard deviation.

**Table 2:** Results of the one-way ANOVA followed by a Bonferroni post hoc test performed to compare blood Phe levels associated with the different treatment-adherence ranges.

Comparison	Estimate	Standard Error	Degrees of Freedom	t Value	Pr > |t|	Adjusted P
75–100% vs. 50–74%	−80.10	69.69	27	−1.15	0.2605	0.7814
75–100% vs. 25–49%	−298.15	82.70	27	−3.61	0.0012	0.0037
50–74% vs. 25–49%	−218.05	92.67	27	−2.35	0.0262	0.0785

**Table 3:** Data on modality, frequency, and timing of daily supplement intake collected from adolescent and adult patients with phenylketonuria.

Id Patient	Modality of Supplement Intake (with)	Frequency of Supplement Intake (Times a Day)	Timing of Supplement Intake					
			Breakfast	Morning Snack	Lunch	Afternoon Snack	Dinner	After Dinner
P1	Water	2	x		x			
P2	Other foods *	2	x	x				
P3	Fruit juice, milk, or milk-based beverage	3	x			x		x
P4	Taken alone	3	x		x		x	
P5	Fruit juice, milk, or milk-based beverage	3	x	x				x
P6	Fruit juice, milk, or milk-based beverage	2	x			x		
P7	Fruit juice, milk, or milk-based beverage	2	x			x		
P8	Fruit juice, milk, or milk-based beverage	2	x			x		
P9	Fruit juice, milk, or milk-based beverage	3	x	x		x		
P10	Fruit juice, milk, or milk-based beverage	1	x					
P11	Taken alone	2	x			x		
P12	Taken alone	4	x		x	x	x	
P13	Taken alone	3	x		x			x
P14	Fruit juice, milk, or milk-based beverage	3	x			x		x
P15	Water	2		x		x		
P16	Water	2	x			x		
P17	Fruit juice, milk, or milk-based beverage	2				x		x
P19	Water	4	x		x	x		x
P19	Fruit juice, milk, or milk-based beverage	2	x					x
P20	Fruit juice, milk, or milk-based beverage	1	x					
P21	Fruit juice, milk, or milk-based beverage	2	x					x
P22	Fruit juice, milk, or milk-based beverage	3	x			x		x
P23	Fruit juice, milk, or milk-based beverage	3	x	x	x			
P24	Fruit juice, milk, or milk-based beverage	2	x			x		
P25	Water	2	x				x	
P26	Water/Fruit juice, milk, or milk-based beverage	1						x
P27	Water	3	x		x			x
P28	Taken alone	3	x			x	x	
P29	Water	3	x			x		x
P30	Other foods/Water	2	x	x				
P31	Other foods/Fruit juice, milk, or milk-based beverage	2	x			x		
P32	Other foods	2			x		x	
P33	Other foods	2	x	x				

* Other foods included fruit, free-protein biscuits, and syrup.

## Data Availability

The data are available upon request from the corresponding author.
